# Early life antibiotic exposure affects pancreatic islet development and metabolic regulation

**DOI:** 10.1038/srep41778

**Published:** 2017-02-02

**Authors:** Jiaying Li, Kaiyuan Yang, Tingting Ju, Tracy Ho, Catharine A. McKay, Yanhua Gao, Shay K. Forget, Stephanie R. Gartner, Catherine J. Field, Catherine B. Chan, Benjamin P. Willing

**Affiliations:** 1 Department of Agricultural, Food and Nutritional Science, University of Alberta, Edmonton, AB, T6G 2P5, Canada; 2 Department of Physiology, University of Alberta, Edmonton, AB, T6G 2H7, Canada

## Abstract

Childhood antibiotic exposure has been recently linked with increased risk of metabolic disease later in life. A better understanding of this association would potentially provide strategies to reduce the childhood chronic disease epidemic. Therefore, we explored the underlying mechanisms using a swine model that better mimics human infants than rodents, and demonstrated that early life antibiotic exposure affects glucose metabolism 5 weeks after antibiotic withdrawal, which was associated with changes in pancreatic development. Antibiotics exerted a transient impact on postnatal gut microbiota colonization and microbial metabolite production, yet changes in the expression of key genes involved in short-chain fatty acid signaling and pancreatic development were detected in later life. These findings suggest a programming effect of early life antibiotic exposure that merits further investigation.

Antibiotics are frequently prescribed to infants and children to prevent bacterial infections, resulting in high childhood antibiotic exposure rates worldwide^1^,^2^,^3^,^4^. However, antibiotic exposure of infants during the first 6^2^,^5^,^6^ to 12 months^7^,^8^ of life has been associated with increased BMI, body mass or risk of overweight up until 12 years of age. Early life, especially the first 6 months after birth is considered as a critical period of gut microbial colonization^9^. During this time, the microbiota is unstable and susceptible to environmental changes^10^,^11^; therefore administration of antibiotics could critically alter the development of the gut microbiota^12^,^13^,^14^,^15^. In addition, physiological differences observed between germ-free and conventional animals^16^,^17^, as well as the metabolic changes after fecal transplantation^18^, suggest a role of gut microbiota in host development and metabolism. Therefore, it can be speculated that an altered gut microbiome during early life may result in changes in host development, leading to metabolic disease later in life^19^,^20^,^21^.

It has been shown that sub-therapeutic antibiotic treatment of C57BL/6 J mice prenatally or at weaning resulted in a changed gut microbiome, increased adiposity and subsequently altered hepatic metabolism of lipid and cholesterol^22^,^23^, which was worsened with a high fat diet^24^. Pulsed therapeutic-dose antibiotic treatment at days 10–15, 28–31 and 37–40 of life affected microbiota composition, accelerated total body mass and bone growth, and changed hepatic gene expression^25^. These findings support the potential role of early-life antibiotic-disrupted microbiota in mediating the development of childhood overweight/obesity, which strongly predicts chronic metabolic diseases such as diabetes in adulthood^26^. However, the mechanisms by which antibiotic-altered microbiome disrupts metabolic regulation are still unclear.

Short chain fatty acids (SCFAs) produced by the gut microbiota have been suggested to play a pivotal role in regulating host metabolism. Their profile and abundance can be significantly changed by altering microbial composition because certain bacteria preferentially produce specific SCFA^27^. Increasing circulating SCFA concentrations by direct administration or enhancing microbial production can improve metabolic outcomes^28^,^29^. Prolonged antibiotic treatment in the adult non-obese diabetic (NOD) mouse restructured gut microbiota composition and resulted in reduced SCFA, leading to accelerated T1D^30^. Conversely, early life sub-therapeutic antibiotic therapy increased adiposity and altered metabolism in mice, which was associated with an increase in SCFA produced by antibiotic-disrupted gut microbiota^22^. In addition, SCFAs are absorbed in the colon and are able to act as signal transduction molecules between microbes and the host. SCFA receptors, G protein-coupled receptor (GPR) 41 and GPR43 have been reported to be expressed in various organs and implicated as mediators of host energy metabolism using knockout mouse models^31^,^32^. They also exert direct effects on pancreatic β-cells by modulating insulin secretion and cell proliferation^33^,^34^,^35^,^36^. Interestingly, in intra-uterine growth retardation (IUGR) models, early perturbation of gut microbiota is associated with hypermethylation of genes key to islet development, such as pancreatic and duodenal homeobox-1 (*PDX-1*)^37^. Similar outcomes might be expected in a host with antibiotic-disrupted gut microbiota.

In the present study, we hypothesize that therapeutic antibiotic treatment in early life elicits gut microbial perturbation, which results in dysregulation of glucose metabolism later on. We tested our hypothesis using a neonatal pig model, due to anatomical and physiological similarities with the human infant and the ability to directly administer antibiotics to the newborn animal. Newborn piglets were sow-fed and co-housed to avoid confounding effects of nutrition and environment. Amoxicillin, a broad-spectrum antibiotic commonly prescribed to infants, was administered from birth to postnatal day (PND) 14 at a therapeutic dose. By adopting this well-controlled animal model that resembles traits of human infants, we aimed to 1) investigate the effects of antibiotic exposure before weaning (day 0 to 14) on metabolic outcomes later in life, 2) examine the impact of antibiotic exposure on pancreatic development, and 3) determine antibiotic-induced changes in microbial composition and metabolism in order to explore the possible mechanisms of early-life antibiotic exposure and metabolic outcomes later in life.

## Results

### Early life antibiotic exposure alters glucose metabolism

**Figure 1:**
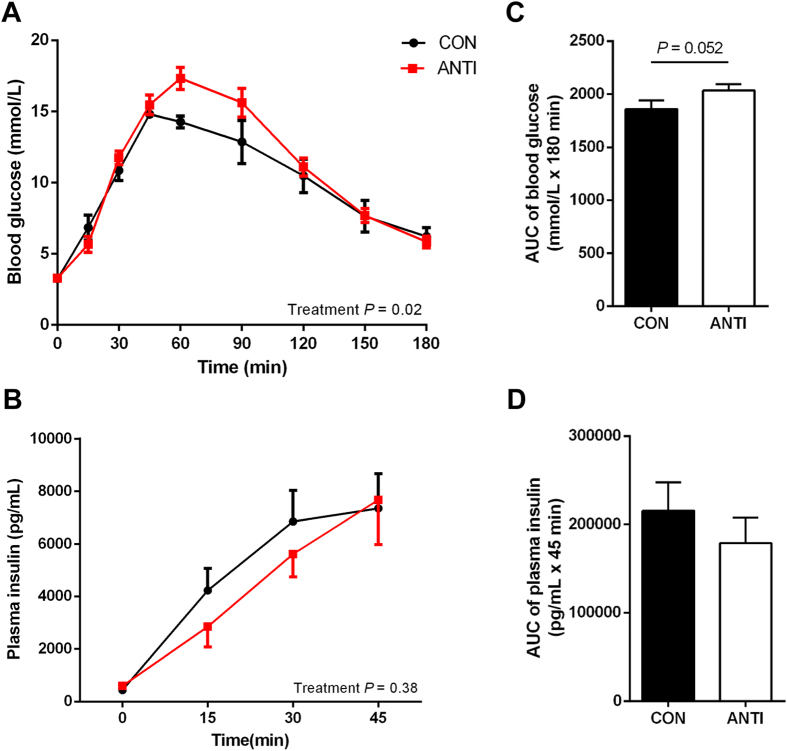
Early life antibiotic exposure alters glucose metabolism later in life. At PND 49, oral glucose tolerance test was conducted to determine the effect of early antibiotic treatment on glucose homeostasis. After overnight fasting, blood glucose (**A**) was measured at 0, 15, 30, 45, 60, 90, 120, 150 and 180 min after consuming 50 g of grounded pregrower fodder mixed with 2 g/kg glucose solution. Plasma insulin (**B**) was measured at 0, 15, 30 and 45 min. Area under the curve (AUC) of glucose response (**C**) and insulin secretion (**D**) were calculated from **A** and **B**, respectively. N = 8 for all groups.

Newborn piglets were treated for two weeks with 30 mg/kg/day of amoxicillin, which is equivalent to the dose often prescribed to human infants. Body weights did not differ significantly between the two groups, both at birth and PND 49 (Supplemental Fig. S1). To examine the effect of antibiotic treatment on glucose homeostasis, responses to oral glucose tolerance test (OGTT) were measured at PND 49. The OGTT was used as an indicator of insulin sensitivity and pancreatic β-cell function. Firstly, we confirmed that after 2-week acclimatization, OGTT procedures caused minimal stress to the experimental animals and did not affect blood glucose levels (data not shown). Fasting blood glucose (FBG) and plasma insulin (FPI) were not different between the two groups (FBG (mmol/L): CON, 3.2 ± 0.05, N = 7; ANTI, 3.3 ± 0.17, N = 8; FPI (ng/mL): CON, 445.0 ± 56.6, N = 6; ANTI, 598.6 ± 120.3, N = 7). In response to glucose challenge, the ANTI group had higher glucose excursion during OGTT compared to CON with no effect of litter (Fig. 1A, *P* < 0.05). Blood glucose concentration in the ANTI group peaked at 60 min and was significantly higher (*P* < 0.05) than that of the CON group. A trend (Fig. 1B, *P* = 0.052) toward increased area under the curve (AUC) of glucose response during OGTT in the ANTI group was also observed. However, plasma insulin concentrations in the first 45 min during the OGTT were quite variable and did not differ significantly between treatment groups (Fig. 1C and D).

### Early life antibiotic exposure alters pancreatic islet function

**Figure 2:**
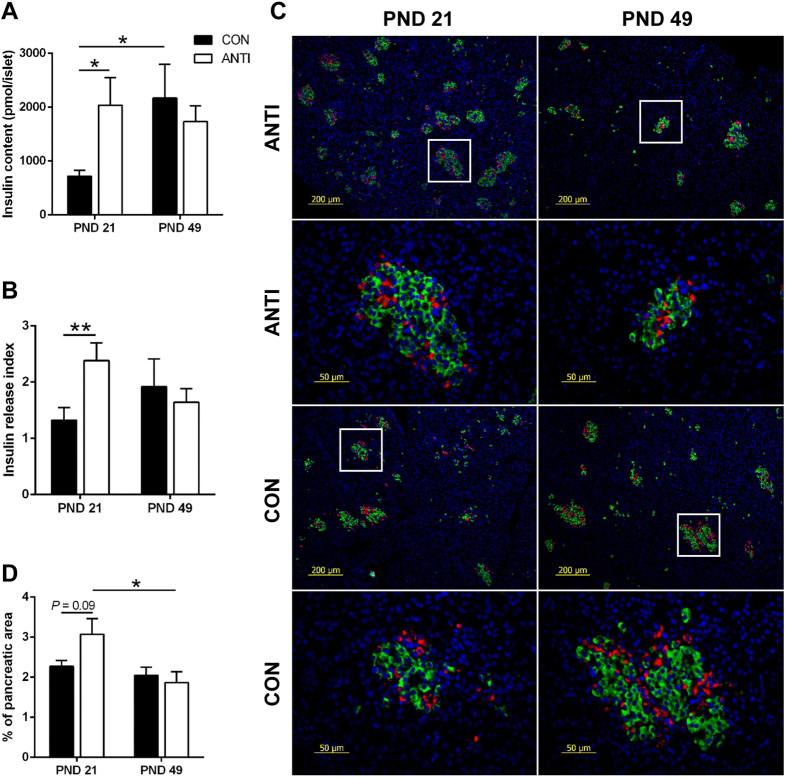
Early life antibiotic exposure leads to changes in pancreatic islet function. Glucose-stimulated insulin secretion (GSIS) was conducted using isolated pancreatic islets. Isolated islets were incubated in DMEM plus 2.8 and 16.5 mmol/L glucose for 90 min. Insulin content (**A**) and insulin stimulation index (**B**) were calculated as described in the methods. (**C**) Representative immunofluorescent staining for insulin (green) and glucagon (red) in paraffin-embedded pancreatic tissue (rows 2 and 4 showing detail from rows 1 and 3, respectively). The percentages of insulin (**D)** positive area versus the total pancreas areas were calculated as estimates of pancreatic β-cell mass. N = 6–7 for each group. *P < 0.05, **P < 0.01.

The insulin responses during OGTT can be affected by multiple factors *in vivo*, therefore, pancreatic islet cell function and β-cell areas were directly assessed to understand the possible causes of reduced glucose tolerance in the ANTI pigs. Glucose-stimulated insulin secretion (GSIS) was conducted using isolated islets from pigs in ANTI and CON groups at PND 21 and 49. At PND 21, isolated islets from ANTI pigs had two-fold higher insulin content compared to CON (Fig. 2A, *P* < 0.05). In addition, islets from ANTI pigs were more responsive to high glucose stimulation as indicated by a higher insulin release index compared to CON (Fig. 2B, *P* < 0.01). However, at PND 49, there was no difference in either insulin content or insulin release index between the two treatment groups. When comparing between ages within the same group, pancreatic islets from CON pigs had higher insulin content (*P* < 0.05) and 45% higher insulin release index at PND 49 compared with PND 21. In contrast, there were no differences in either insulin content or insulin release index between the two ages in ANTI pigs.

We then quantified pancreatic β-cell area at PND 21 and 49. Formalin-fixed samples were sectioned and stained for insulin (β-cell) and glucagon (α-cell). Representative immunofluorescent staining sections are shown in Fig. 2C. Compared to CON pigs, ANTI pigs had a non-significant higher (Fig. 2D, *P* = 0.09) fractional β-cell area at PND 21, which agreed with the GSIS result, that ANTI pigs at PND 21 had higher insulin content in isolated islets. However, at PND 49 β-cell area decreased significantly (Fig. 2D, *P* < 0.05) compared to PND 21 in ANTI pigs, whereas, CON pigs had similar β-cell areas at PND 21 and 49 (Fig. 2D).

### Early life antibiotic exposure alters pancreatic islet development

**Figure 3:**
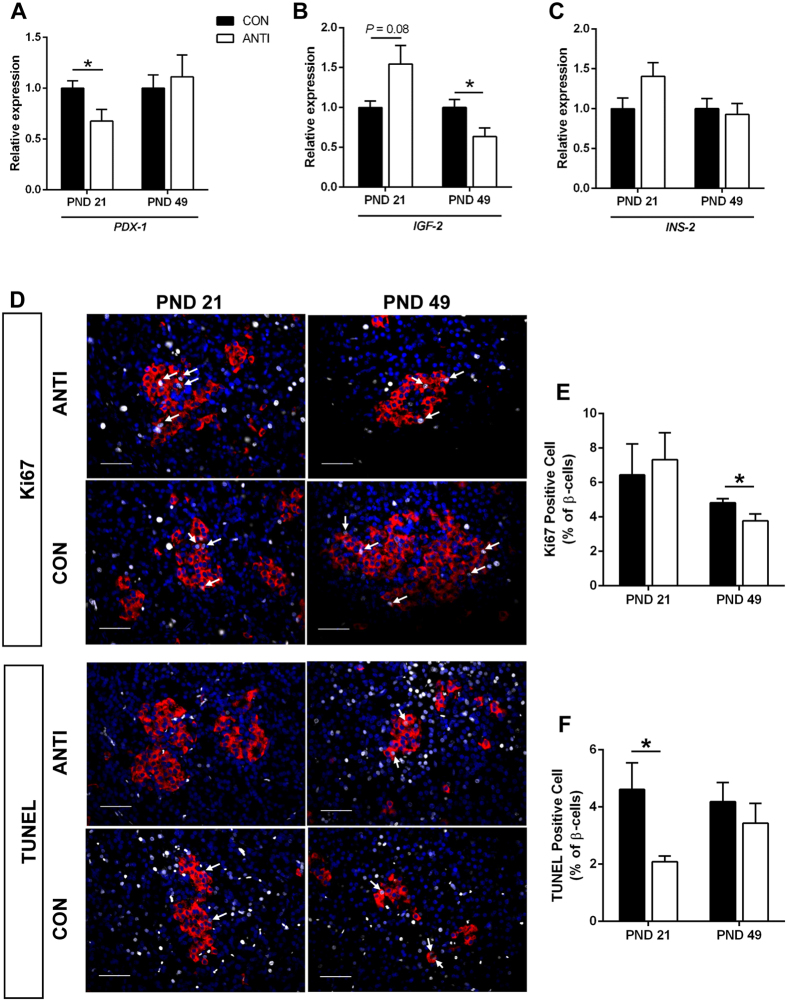
Early life antibiotic exposure leads to changes in pancreatic islet development. The relative expression of *PDX-1* (**A**), *IGF-2* (**B**) and *INS-2* (**C**) were measured by RT-qPCR in whole pancreas at PND 21 and PND 49. (**D**) Representative immunofluorescent staining for Ki67 and TUNEL (grey), insulin (red) and DAPI (blue) in paraffin-embedded pancreatic tissue. Ki67- and TUNEL-positive nuclei were indicated by white arrows, scale bar = 50 μm. The ratios of Ki67 (**E**)-/TUNEL (**F**)-positive cells vs insulin-positive cells were calculated. N = 6–7 for each group. *P < 0.05.

To further explore the potential mechanisms that led to the functional and morphological changes seen in β-cells, genes involved in pancreatic development, insulin secretion and β-cell function were analyzed by reverse transcription quantitative polymerase chain reaction (RT-qPCR) in whole pancreas from both groups at PND 21 and 49. *PDX-1* is a transcription factor that plays an indispensable role in the development of both endocrine and exocrine pancreas, as well as in β-cell differentiation and function^38^. Our results showed that at PND 21, relative *PDX-1* mRNA levels in the pancreas of the ANTI pigs were 30% lower than that of CON (Fig. 3A, *P* < 0.05). This difference had disappeared at PND 49 (Fig. 3A). We also measured mRNA expression of insulin-like growth factor (*IGF)-2*, which is involved in β-cell regeneration and proliferation^39^ and has been reported to be maternally imprinted and epigenetically regulated in newborns exposed to antibiotics^40^. Pancreas from ANTI pigs had 1.5-fold higher *IGF-2* expression compared to CON at PND 21 (Fig. 3B, *P* = 0.08). In contrast, at PND 49, expression of *IGF-2* was lower in ANTI pigs than CON (Fig. 3B, *P* < 0.05). There was no difference in *INS* expression between the two groups at PND 21 and PND 49 (Fig. 3C).

Additionally, to estimate the rates of β-cell proliferation, pancreatic slides were stained for Ki67, a protein present during all active phases of the cell cycle (G1, S, G2, and mitosis), but absent from resting cells (G0)^41^. The proportion of Ki67-positive vs total β-cells was significantly lower (P < 0.05) in ANTI than CON islets at PND 49, but not different at PND 21 (Fig. 3E). β-cell apoptosis rate was measured by TUNEL, a method for detecting apoptotic DNA fragmentation by labeling the terminal end of nucleic acids^42^. ANTI pigs at PND 21 had a ~50% decrease (P < 0.05) in apoptotic β-cells compared to CON, whereas no difference at PND 49 (Fig. 3F). Together these results indicate that islet development was affected by early life amoxicillin treatment.

We also explored whether inflammatory responses in pancreas were affected by early life antibiotic treatment; however, gene expression of cytokines and chemokines in pancreas at PND 21 and PND 49 was not markedly affected by antibiotic-altered gut microbiota (Supplemental Fig. S6)

### Early life antibiotic exposure transiently alters gut microbial composition

**Figure 4:**
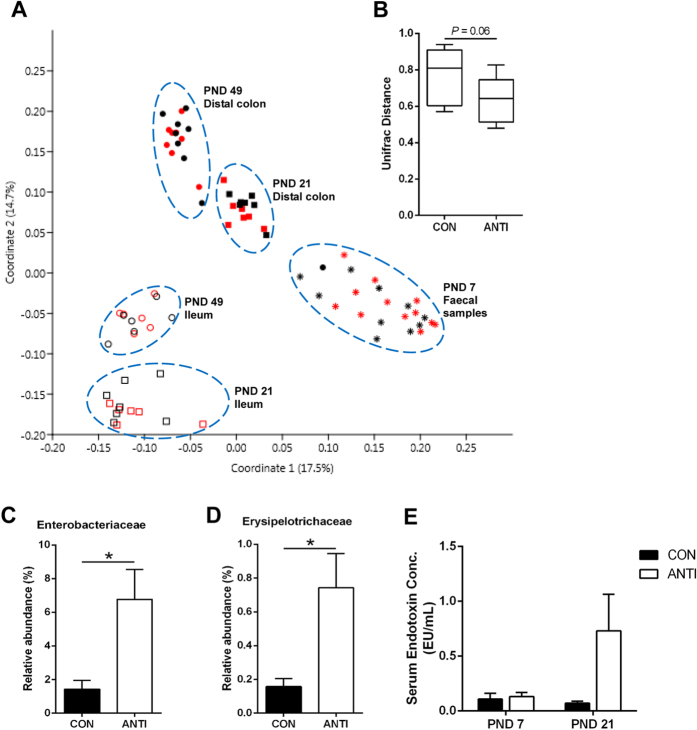
Early life antibiotic exposure alters gut microbial composition. (**A**) PCoA plot of bacterial community composition in amoxicillin-treated (ANTI, red) and control (CON, black) pigs using Bray-Curtis dissimilarity metrics at the OTU level. All OTUs were retained in the analysis. Faecal samples and ileal and distal colon contents were plotted as follow: PND 7 faecal samples (star, n = 11), PND 21 ileum (hollow square, n = 6), PND 49 ileum (hollow circle, n = 6), PND 21 distal colon (full square, n = 7) and PND 49 distal colon (full circle, n = 8). All OTUs were retained in the analysis. (**B**) Beta diversity within group for ANTI and CON pigs at PND 7. Relative proportion of (**C**) Enterobacteriaceae and (**D**) Erysipelotrichaceae at PND 7. (**E**) LPS concentrations (serum endotoxin) was measured in serum from pigs at PND 7, 14 and 21. N = 6–7 for each group, *P < 0.05.

To examine both short- and long-term effects of amoxicillin on gut microbiota, we measured gut microbial composition in pigs at different ages. DNA was extracted from fecal samples at PND 7, and ileal and distal colonic content at PND 21 and PND 49. The extracted DNA was subjected to 454 pyrosequencing, yielding 1907 ± 86 sequences per sample that passed quality filtration. Microbial data were analyzed at the community level and at each taxonomic level. There was a significant effect of sampling location (ileum vs distal colon) and sampling time (PND 7, 21 and 49) on community composition (*P* < 0.001), as visualized on a principle coordinate analysis (PCoA) plot (Fig. 4A and Supplemental Fig. S2). However, the overall community composition was not significantly affected by treatment at any time point as indicated by analysis of molecular variance (AMOVA) (D7: *P* = 0.75, D21: *P* = 0.27, *D49: P* = 0.85). There was a trend for reduced beta diversity in the ANTI pigs compared to CON at day 7 (Fig. 4B, *P* = 0.06). However, there was no effect of antibiotic treatment on alpha diversity at any time point (Supplemental Fig. S3). While no global changes were observed between treatments, a substantial increase in the family Enterobacteriaceae was observed in ANTI pigs compared with CON at PND 7 (Fig. 4C, P < 0.05), which was largely represented by a single operational taxonomic unit (OTU) classified as Escherichia (6.0% and 1.2%). Erysipelotrichaceae was also increased in ANTI pigs compared to CON at PND 7 (Fig. 4D, *P* < 0.05). The final notable change in microbial population at PND 7 in the ANTI group was an increase in an OTU identified as *Mitsuokella jalaludinii*. It was detected in 8 out of 11 samples in ANTI group and represented a mean of 0.17% of 16 S rRNA gene reads, however it was undetectable in all CON samples. Importantly, all of the differences observed at PND 7 disappeared after antibiotic treatment ended (PND 21 and 49, Supplementary Fig. S4). The shift in microbial composition observed did not lead to significant changes in serum lipopolysaccharide (LPS) concentrations at PND 7 and 21 (Fig. 4E).

### Early life antibiotic exposure affects SCFA metabolism and signaling

**Figure 5:**
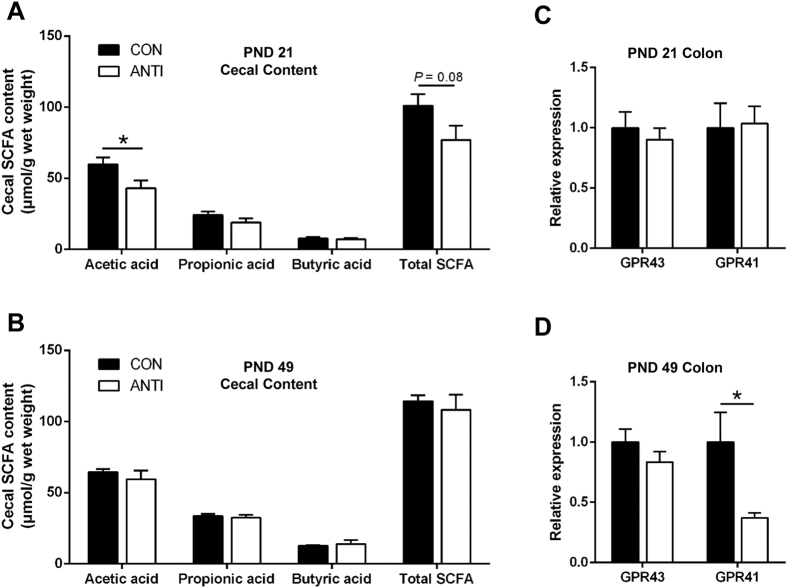
Early life antibiotic exposure affects SCFA metabolism and signaling in the intestine. Short chain fatty acid (SCFA) concentrations were measured in cecal contents at PND 21 (**A**) and 49 (**B**). The relative gene expression of *GPR41* and *GPR43* were measured in colon samples at PND 21 (**C**) and 49 (**D**). N = 6–7 for each group, *P < 0.05.

Intestinal microbial alteration could lead to changes in SCFA metabolism; therefore, we measured SCFA concentrations in cecal content from pigs at PND 21 and 49. At PND 21, ANTI pigs had lower acetate concentration (Fig. 5A, *P* < 0.05) and showed a trend for lower total SCFA (Fig. 5A, *P* = 0.08) compared to CON pigs. However, at PND 49, the differences between the two groups disappeared (Fig. 5B). To further examine the effects of antibiotic exposure on SCFA signaling, expression of SCFA receptors, *GPR41* and *GPR43*, were measured in colon tissue from pigs at PND 21 and 49. There was no difference in *GPR41* and *GPR43* expression between the two treatment groups at PND 21 (Fig. 5C); however, at PND 49 *GPR41* expression was 60% lower in the colon of ANTI pigs (Fig. 5D *P* < 0.05).

## Discussion

There is growing interest in the role of the gut microbiota in the global pandemic of obesity and type 2 diabetes (T2D). The gut microbiota matures with the host and is actively involved in host metabolism and development^43^. Several epidemiological studies have correlated antibiotic exposure in infancy with increased risk of metabolic disease later in life^2^,^5^,^44^, emphasizing the importance of early life gut microbial alteration in health outcomes. As antibiotics will continue to be a key tool required to support infant health, it is crucial to understand how early life antibiotics contribute to chronic diseases. In the present study, we used a porcine model of the human infant to unveil a possible missing link between early life antibiotic exposure and metabolic outcomes. This is the first study to provide experimental evidence that therapeutic antibiotic exposure in early life results in altered pancreatic islet function and development. Furthermore, these changes in host metabolism were correlated with altered microbial composition and activity in early life and subsequent capacity for SCFA induced responses. It is worth noting that the effect of antibiotics on microbiota was only seen in pigs at PND 7 and ceased after antibiotic withdraw, yet, the impact on glucose tolerance was observed at PND 49, suggesting a possible programming effect of the early life gut microbiota. Further studies into adulthood will be necessary to determine whether these effects persist through life.

Pigs that received therapeutic amoxicillin exposure at birth through PND 14 had reduced glucose tolerance at PND 49, even though their β-cell area and islet function did not differ from control pigs at this age. Insulin response during the first 45 minutes didn’t show differences, with the limitation that later time points were missing. However, amoxicillin treatment did alter pancreatic islet development. Pigs treated with antibiotics had hyper-developed (or early matured) pancreatic islets (increased insulin content and insulin release index compared to control pigs) already at PND 21, followed by a significant decrease in β-cell area from PND 21 to PND 49. In contrast, isolated islets from control pigs saw increased insulin content over this same period. It has been appreciated that substantial remodeling of the endocrine pancreas occurs in neonatal life. A transient decrease in β-cell growth rate due to a wave of progressive apoptosis has been related with weaning, as a result of β-cells adapting to the increased carbohydrates in the diet^45^,^46^,^47^. Even though β-cells are highly dynamic and resilient, and can compensate to cope with increased insulin demand when challenged with high glucose^48^, the low self-replication capacity in adults makes β-cells unable to regenerate significantly following extensive tissue injury or chronically increased metabolic demands^49^. Therefore, perturbations during the period of β-cell development and maturation could result in lasting long-term consequences. The changes observed in the current study suggest β-cells in ANTI pigs had a delayed apoptosis period compared to CON, which may have contributed to the glucose intolerance in ANTI pigs at PND 49. Indeed, quantification of β-cell proliferation and apoptosis rates showed that at PND 21 β-cells from ANTI pigs featured significantly lower apoptosis rates, yet similar proliferation rates compared to CON. Furthermore, at PND 49 ANTI pigs, which already had slightly reduced glucose tolerance, had decreased β-cell proliferation rates, suggesting impaired ability to compensate for insulin demand. At PND^49^, the pig and pancreas are still developing, therefore it is yet unclear whether the early life antibiotic exposure alters mature pancreatic function and glucose tolerance in adulthood. Further studies conducted in piglets fed high fat diet to stress the islets might exacerbate the metabolic phenotype.

In addition, assessment of *PDX-1* and *IGF-2* mRNA expression in pancreas further supported the disrupted β-cell development seen in ANTI pigs. *PDX-1* plays a central role in β-cell function and survival^38^. In our study, at PND 21, *PDX-1* expression was lower in the ANTI pigs, suggesting the beginning or ongoing regression of β-cell function and/or mass, even though they had highly functioning islets and increased β-cell area compared to CON. It has been reported that pancreatic islets from T2D patients have lower expression of *PDX-1*^50^. In IUGR rats, which also develop impaired insulin secretion, *PDX-1* mRNA level was reduced by 50%^37^. At PND 49, expression of *PDX-1* had normalized compared to CON, which was in agreement with the observation that β-cell function and area were not different between the two groups. In contrast, mRNA expression of *IGF-2* tended to be higher at PND 21 while lower at PND 49 in ANTI compared to CON. As discussed above, pancreatic islets undergo postnatal remodeling, in which β-cell apoptosis and neogenesis play a significant role^45^. The timing of increased β-cell apoptosis is associated with a loss of *IGF-2* expression^51^. IGF-2 is a major growth factor that is highly expressed during fetal growth and progressively down-regulated after birth^52^. IGF-2 is able to promote pancreatic islet survival against apoptotic stimuli^53^. However, it was recently shown that overexpression of *IGF-2* in β-cells could lead to β-cell dysfunction and make β-cells prone to damage^54^. Therefore, a trend for increased postnatal *IGF-2* expression seen in antibiotic treated pigs at PND 21 may increase β-cell area by promoting proliferation/neogenesis, yet it could also cause potential β-cell damage. This could contribute to the substantial loss of β-cell mass accompanied by reduced glucose tolerance in ANTI pigs at PND 49. Overall, the gene expression of *PDX-1* and *IGF-2* coincided with the changes in islet/β-cell function and β-cell area. All of these results indicate a programming effect of early life antibiotics on pancreatic development.

We also investigated whether inflammation might be involved in differences in glucose metabolism and pancreatic development. However, pancreatic gene expression of cytokines and chemokines, including TNF-α, IFN-α, IL-6, IL-8, CXCL-2, CXCL-10, and CCL-2, were not affected by antibiotic treatment (Supplemental Fig. S6) at the time points they were measured.

To explore how antibiotics might alter metabolic phenotypes, we examined the microbial composition in the gut. Similar to previous studies^22^,^55^, antibiotic treatment did not cause significant global change in microbiota, however, an increase in the abundance of Enterobacteriaceae and Erysipelotrichaceae was observed in ANTI pigs after 7-day antibiotic treatment. Many members of Enterobacteriaceae carry resistance genes including those that encode extended-spectrum β-lactamases, which attack β-lactam ring^56^. This may explain why treatment with amoxicillin, a member of the β-lactam antibiotic family, specifically increased the abundance of Enterobacteriaceae. Elevated Enterobacteriaceae abundance has previously been correlated to obesity development^57^. Enterobacteriaceae is a large family of gram-negative bacteria, consisting of both commensals and pathogens that contribute to the enteric pool of lipopolysaccharide (LPS), which plays a significant role in mediating host insulin resistance^58^. However, serum LPS concentrations were not changed at PND 7 despite the increase in Enterobacteriaceae in ANTI pigs. It should be noted that data from the LPS assay was quite variable and may warrant further investigation. Intestinal alkaline phosphatase (IAP) detoxifies bacterial LPS^59^,^60^ and IAP gene expression has previously been shown to be induced by circulating LPS^61^. In a pig model for *in utero* antibiotic exposure, ileal IAP concentration was transiently reduced in offspring at PND 14^62^. Administration of IAP was able to prevent the later development of metabolic syndrome in mice treated with early-life antibiotics^63^. However, in the present study we found no difference in IAP gene expression (Supplemental Fig. S5A), but a trend for reduced IAP activity in ANTI pigs at PND 49 (Supplemental Fig. S5B, *P* = 0.08). Similar to Enterobacteriaceae, increased abundance of Erysipelotrichaceae has been observed in a diet-induced obesity model^64^ and a decrease was observed after gastric bypass surgery in obese individuals^65^, suggesting this bacteria family may be involved in obesity development, although no mechanism has been postulated.

Despite only modest changes in microbial composition, significant changes in SCFA production and signaling were observed. Specifically, both total SCFA and acetate production was reduced in ANTI compared to CON at PND 21. SCFA have been shown to protect against diet-induced obesity by stimulating gut hormones including glucagon-like peptide-1 (GLP-1) secretion via the activation of GPRs^28^,^66^,^67^. GLP-1 is known to inhibit β-cell apoptosis and stimulate insulin secretion, β-cell proliferation and differentiation^38^,^68^. Therefore, a lower production of SCFA could affect GLP-1 secretion negatively, thereby altering β-cell development. Consistent with our results, a previous study showed that a single high dose of streptomycin (20 mg per mouse) caused reduction in acetate, propionate, and butyrate of the cecum from 7-week old mice after 72 hours^69^. Whereas a standard dose of metronidazole for 7 days eliminated fecal propionate production in a 3-year old child with propionic acidemia^70^. However, in a mouse model, sub-therapeutic doses of antibiotics (1 mg antibiotics per gram body weight of mouse) prenatally resulted in increased SCFA production^22^. The contradictory results could be due to the differences in animal model and antibiotic dosage. Reduced *GPR41* expression was seen in ANTI pigs at PND 49, however relatively little is known on how the expression of *GPR41* in the intestine affects host response. Expression of *GPR41* has previously been positively correlated with SCFA concentrations in goats^71^. Furthermore, it was recently shown that in obese and diabetic patients, there was a significant negative correlation between body mass and *GPR41* methylation^66^. Although still unclear, it is plausible that reduced *GPR41* expression could play a role in the observed antibiotic induced changes in response to glucose challenge.

In summary, by using a swine model for human infants, we provide direct evidence that early life antibiotic exposure leads to impaired glucose metabolism later in life. Gut microbiota and metabolite production were transiently affected by antibiotics, yet changes in the expression of key genes involved in SCFA signaling and pancreatic development were detected in later life, indicating a programming effect of antibiotic exposure. Furthermore, amoxicillin treatment was associated with changes in pancreatic development, which could contribute to the observed changes in glucose tolerance between the two groups. Taken together, our work in this animal model provides experimental evidence and a possible mechanism for the epidemiological evidence that early life antibiotics impact on host metabolism later in life.

## Methods

### Animal maintenance

This study was performed according to the guidelines provided by Canadian Council on Animal Care using protocols approved by the University of Alberta Animal Care and Use Committee. Pigs were managed according to approved protocols at the Swine Research and Technology Centre (SRTC), University of Alberta.

Four litters of crossbred piglets (Duroc × Large White/Landrace) born from 2^nd^ or 3^rd^ parity sows, were randomized to antibiotic (ANTI) and control groups (CON). Amoxicillin (30 mg/kg/day) or placebo (antibiotic flavoring) were administered by oral feeding twice daily (8 am and 4 pm) at birth through PND 14. Body weights were recorded weekly.

At PND 21, 8 pigs from each treatment were terminated for sample collection. The remaining pigs were weaned, penned by litter in a nursery room, and fed with a standard phase-feeding program. At PND 49, another 8 pigs from each treatment were terminated. All animals were maintained on a 12-hour light-dark cycle with room temperature of 22–25 °C and allowed free access to food and water. Ingredients of the piglet diets are shown in Supplemental Table S1.

### Oral glucose tolerance test

Two weeks prior to OGTT, pigs were introduced to a standard meal consisting of 50 g ground pregrower fodder, and acclimated to handling and standing in a sling. OGTT was conducted after overnight fasting. An hour before OGTT, ears were cleaned and Lidocaine/prilocaine cream applied (EMLA Cream; AstraZeneca, Mississauga, Ontario) to reduce pain. After measuring fasting blood glucose (FBG) animals were given the standard meal (50 g) mixed with 2 g/kg glucose solution. Upon finishing the meal (time 0), glucose was measured with a glucometer (Accu-Check Compact Plus; Roche Diagnostics) in whole blood from ear vein at 15, 30, 45, 60, 90, 120, and 180 min. Additional blood samples were collected - during the first 45 min by 70 μl microhematocrit capillary tubes coated with ammonium heparin (Fisher Scientific). Blood samples were centrifuged at 1500 rpm for 10 min at 4 °C and plasma collected and stored at −80 °C until assayed for insulin by ELISA, according to the manufacturer’s instructions (Alpco Diagnostics, Salem, N.H., USA). Blood glucose and plasma insulin concentrations were plotted as a function of time, and area under the curve was calculated using established methods^72^.

### Animal euthanasia and sampling

At PND 21 and 49, two pigs per treatment per litter were euthanized in the morning (between 0700–1100) for sample collection. Blood samples were collected from jugular vein, and serum samples were aseptically collected, snap frozen in liquid nitrogen, and stored at −80 °C for LPS assay. Pancreas was collected for pancreatic islets isolation [stored in ice-cold Hanks’ Balanced Salt Solution (HBSS; Sigma-Aldrich Canada Ltd., Oakville, ON, Canada) supplemented with 0.2% bovine serum albumin (BSA), 25 mM HEPES, 100 mg/L L-glutamine and 0.35 g/L NaHCO_3_], immunohistochemistry (10% formalin, Fisher Scientific) and RNA extraction (snap frozen in liquid nitrogen). The entire intestine was subsequently extracted. The ileum (20 cm proximal to the ileo-cecal junction), proximal colon and distal colon were carefully dissected; and mucosal samples were collected, immediately snap-frozen in liquid nitrogen and stored at −80 °C. Ileal, cecal and distal colon content were aseptically collected and snap frozen in liquid nitrogen until storage at −80 °C. In addition, at PND 7, fecal samples were collected before tissue collection using sterile dry swabs (BD Falcon™ SWUBE™, BD Canada, Mississauga, ON) and stored in −80 °C.

### Pancreatic islet isolation and culture

Islets of Langerhans were freed from pancreas tissues after a two-time digestion (35 and 20 mins, respectively) in collagenase solution (1 mg/mL in HBSS + 0.2% BSA) at 37 °C, 130 rpm. Samples were then washed with HBSS + 0.2% BSA and filtered through 160 μm nylon filter. Each pellet sample was enriched for islets by means of a 27%, 23%, 14% dextran density gradient centrifuged at 1,500 rpm for 15 min at room temperature. Islets were harvested from the 23% gradient layer, washed and resuspended in HBSS + 0.2% BSA, and separated from exocrine tissue by hand picking under a dissection microscope. Islets were cultured overnight in CMRL1066 medium (Sigma) supplemented with 0.5% BSA at 37 °C in humidified air containing 5% CO_2_.

### Glucose-stimulated insulin secretion

Cultured islets were washed with Dulbecco’s modified Eagle’s medium (DMEM; Gibco, Burlington, ON, Canada) with 0.1% BSA. To measure insulin release, quadruplicate samples of 3 islets/vial were incubated in DMEM with 2.8 and 16.5 mM glucose for 90 min at 37 °C gassed with 95% O_2_ and 5% CO_2_. Supernatants were retained and insulin remaining in the islets was extracted with 3% acetic acid, and then stored at −20 °C for insulin radioimmunoassay (RIA)^73^. Total islet insulin content was calculated by adding insulin secreted into supernatant plus the remaining in the islet pellet, as determined by RIA. From this, the percentage of total insulin secreted was calculated for each data point to eliminate variance caused by islet size. Insulin release index was calculated as the ratio of insulin percentage release in response to 16.5 mM glucose versus 2.8 mM glucose.

### Immunohistochemistry

Pancreas tissues were fixed in 10% phosphate buffered formalin for 24 h. The tissues were dehydrated and embedded in paraffin using standard procedures, and 5 μm sections cut and affixed to glass slides. After dewaxing and rehydration, pancreas sections were treated overnight at 4 °C with primary antibodies: guinea pig anti-insulin (Dako, Burlington, ON, CA), rabbit anti-glucagon (Millipore, Billerica, MA, USA) and rabbit anti-Ki67 (Abcam, Toronto, ON, CA), followed by a 2-hour incubation at room temperature with secondary antibodies: goat anti-guinea pig (IgG H&L, Alexa Fluor^®^ 488; Invitrogen, Burlington, ON, CA) and goat anti-rabbit (IgG H&L, Alexa Fluor^®^ 594; Invitrogen), respectively. All antibodies were diluted to 1:200 in PBS. Slides were then mounted with ProLong^®^ Gold Antifade Mountant with DAPI (Invitrogen). TUNEL staining was performed using *In Situ* Cell Death Detection Kit following manufacturer instructions (Sigma-Aldrich Canada Co., Oakville, ON, CA), and double stained with insulin as described above.

Micrographs of pancreas sections were captured using an Axiovert microscope equipped with Axiovision 4.7 software (Carl Zeiss Microscopy GmbH, Jena, Germany). Pancreatic β-cell area was expressed as the percentage of insulin-positive area vs the entire pancreas area (excluding large ducts and veins) mounted on the slide, which was quantified using ImageJ.

For Ki67 and TUNEL staining, an EVOS FL Auto Cell Imaging System (Thermo Fisher Scientific) was used to capture 30–40 randomly selected sections within the pancreas area. The number of nuclei of the insulin-positive areas and Ki67 and TUNEL positive cells within each of the insulin-positive areas was counted using Image J. The proportions of Ki67 and TUNEL positive β-cells relative to total β-cells were used to estimate the rates of proliferation and apoptosis.

### Microbial composition analysis

Total DNA from fecal samples and ileal and distal colon contents was extracted with the QIAamp DNA Mini Stool Kit (Qiagen, Inc. Mississauga, ON, Canada) following the manufacturer’s instructions, with the addition of a bead beating step (FastPrep instrument, MP Biomedicals, Solon, OH). DNA concentrations and quality were determined by a NanoDrop 2000c. Extracted DNA was diluted to 20 ng/μl for PCR amplification.

The hypervariable regions (V1 to V3) of the bacterial *16 S rRNA* gene were amplified with nucleotide barcoded primer pairs 27 F: 5′-AGAGTTTGATCMTGGCTCAG-3′ and 519 R: 5′-GWATTACCGCGGCKGCTG-3′. The forward primer contained Roche/454 Titanium adaptor A (CCATCTCATCCCTGCGTGTCTCCGACTCAG) and unique 10-bp barcodes, and the reverse primer contained adaptor B (CCTATCCCCTGTGTGCCTTGGCAGTCTCAG). The PCR was performed on an S1000 Thermal Cycler (Bio-Rad, Hercules, CA, USA) using the following parameters: initial denaturation at 98 °C for 1 min, followed by 35 cycles of 98 °C for 10 s, 59 °C for 30 s and 72 °C for 30 s, with a final extension at 72 °C for 7 min. Then triplicate DNA amplification products were mixed and gel-purified (QIAquick gel extraction kit, Qiagen, Valencia, CA). Each amplicon (100 ng) was pooled and pyrosequenced using a 454 Titanium platform (Roche, Branford, CT).

Sequence data that passed Roche’s quality thresholds were processed according to the mothur 454 SOP^74^,^75^ accessed on June 16, 2015. Barcodes were trimmed and quality sequences were obtained by removing sequences containing ambiguous bases and quality read length < 200 bases. Sequences passing quality filter were aligned to the silva bacterial reference alignment. Sequences were clustered based upon 0.97 similarity using UClust into operational taxonomic units (OTUs) and hypothesis testing were performed with normalized data in mothur. Differences in overall community were tested by multivariate analysis of variance (MANOVA) with Bonferroni correction for multiple comparisons. Abundance of bacterial OTUs to phyla was compared using the Mann-Whitney *U*-test or student’s T-test. Inverse Simpson diversity index was used to ascertain differences in alpha diversity based on antibiotic exposure status. Weighted UniFrac distance matrices were calculated for beta diversity analyses.

### Serum LPS assay

Serum LPS concentrations were measured by PYROGENT™–5000 Kinetic Turbidimetric LAL Assay (Lonza, Burlington, ON, CA), according to the manufacturer’s instructions.

### SCFA measurement

SCFA concentrations in cecal content were analyzed by gas chromatography. One gram of distal colonic content was mixed with 4 ml of 25% phosphoric acid, vortexed thoroughly and centrifuged at 3,500 rpm for 10 min at 4 °C. Supernatant was transferred to 1.5 ml tubes and centrifuged at 15,000 rpm for 10 min at 4 °C. The supernatant was then filtered through 0.45 μm filters (Fisher), and mixed with internal standard solution (24.5 mmol/L isocaproic acid) at a ratio of 4:1. Samples were injected into a Stabilwax-DA column (30 m × 0.53 mm i.d. × 0.5 μm film thickness; Restek Corporation, Bellefonte, PA) on a Varian gas chromatograph (Model 3800; Varian Analytical Instruments, Palo Alto, CA) using an autosampler (Model 8400; Varian Inc., Walnut Creek, CA). Samples were run at a split vent flow of 20 mL/min with a column temperature gradient as follows: 80 °C held for 0.1 min, increased to 210 °C at 45 °C/min and held for 5 min at 210 °C. The temperature of the injector and the detector was 250 °C. Peaks were analyzed using Galaxie Software (Varian Inc., Palo Alto, CA) and concentrations of each SCFA were calculated. Total SCFA concentration was the sum of concentrations of the detected SCFA. Final results were normalized by the weight of each sample used.

### RNA isolation and cDNA synthesis

Total RNA was extracted from the pancreas, ileum and proximal colon using the GeneJET RNA Purification Kit (Thermo Scientific). On column DNase (RNase-free DNase set; QIAGEN) was applied to eliminate genomic DNA contamination in the RNA samples. RNA concentrations and purity were determined by a NanoDrop 2000c (Thermo Scientific) RNA quality was further verified with gel electrophoresis (RNA loading dye, Thermo Scientific)^76^. Extracted RNA (1 μg) was then reverse transcribed using Maxima First Strand cDNA Synthesis Kit (Thermo Scientific).

### Reverse transcription quantitative PCR

Gene expression was measured by RT-qPCR using PerfeCTa SYBR^®^ Green SuperMix Rox (Quanta Biosciences Inc., Gaithersburg, MD) on a StepOnePlus real-time PCR System equipped with StepOne software v2.3 (Applied Biosystems, ON, Canada). A two-step thermal cycling protocol was performed as follows: initial denaturation for one cycle at 95 °C for 3 min, followed by 40 cycles at 95 °C for 10 s, and annealing or extension temperature for 30 s. Samples were analyzed in duplicates and glyceraldehyde phosphate dehydrogenase (*GAPDH*) was used as the internal control. Relative gene expression was calculated using the comparative CT (2^−ΔΔCT^) method. Primers were designed in Beacon Designer 7.9 using sequences obtained from the ENSEMBL pig database (Supplemental Table S2), and the amplification products and annealing temperature were tested. Specificity of the amplification was verified by melt curve analysis and evaluation of efficiency (90–110%) of qPCR amplification and no reverse transcriptase control (NRT) as negative control for DNA contamination.

### Intestinal alkaline phosphatase (IAP) activity

Please see the supplemental methods for full details.

### Statistical analysis

Statistical analysis for the randomized block design with two treatments was carried out using SAS (version 9.3; SAS Institute Inc. Cary, NC) and GraphPad Prism v6.02 (La Jolla, CA). Comparisons between two treatment groups were analyzed using a proc-mixed model after normality tests unless otherwise stated. Classification variables included animal, treatment and litter. Treatment was included in the model as a fixed class variable, with litter as a random class variable. Differences between treatments means were determined by utilizing lsmeans treat litter/pdiff stderr statement. For OGTT and body weight data, classification variables included animal, treatment, litter and time. Treatment was included in the model as a fixed class variable, litter was included in the model as a random class variable and time was considered additionally as the experimental unit, and differences between treatments were determined by utilizing lsmeans treat time treat*time/pdiff statement. Data are expressed as mean ± SEM. Statistical significance was expressed as ***P < 0.001, **P < 0.01, and *P < 0.05. A *P* value between 0.1 and 0.05 was considered as a trend.

## Additional Information

**How to cite this article**: Li, J. *et al*. Early life antibiotic exposure affects pancreatic islet development and metabolic regulation. *Sci. Rep.* **7**, 41778; doi: 10.1038/srep41778 (2017).

**Publisher's note:** Springer Nature remains neutral with regard to jurisdictional claims in published maps and institutional affiliations.

## Supplementary Material

Supplemental Information
