# Supplementary material for: Early life antibiotic exposure affects pancreatic islet development and metabolic regulation
Source: Sci Rep. 2017 Feb 2;7:41778. doi: 10.1038/srep41778 (PMC5288777; doi:10.1038/srep41778)
Supplement: Supplemental Information [file srep41778-s1.pdf]

# Early life antibiotic exposure affects pancreatic islet development and metabolic regulation

Jiaying Li [1, †], Kaiyuan Yang [1, †], Tingting Ju [1], Tracy Ho [1], Catharine A. McKay [1], Yanhua Gao [1], Shay K. Forget [1], Stephanie R. Gartner [1], Catherine J. Field [1], Catherine B. Chan [1, 2] and Benjamin P. Willing [1, *]

[1] Department of Agricultural, Food and Nutritional Science, University of Alberta, Edmonton, AB, T6G 2P5, Canada

[2] Department of Physiology, University of Alberta, Edmonton, AB, T6G 2H7, Canada

* Corresponding author willing@ualberta.ca

† These authors contributed equally to this work

**Supplemental information**

**Supplemental Figures**

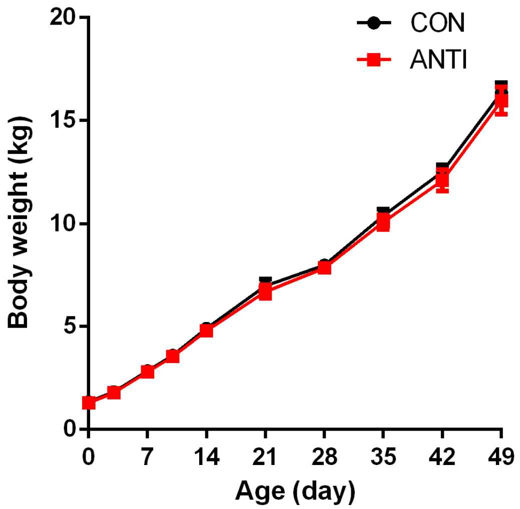


**Supplemental Figure S1. Effect of postnatal antibiotics on body weight.**

ANTI, N = 16; CON, N=14.

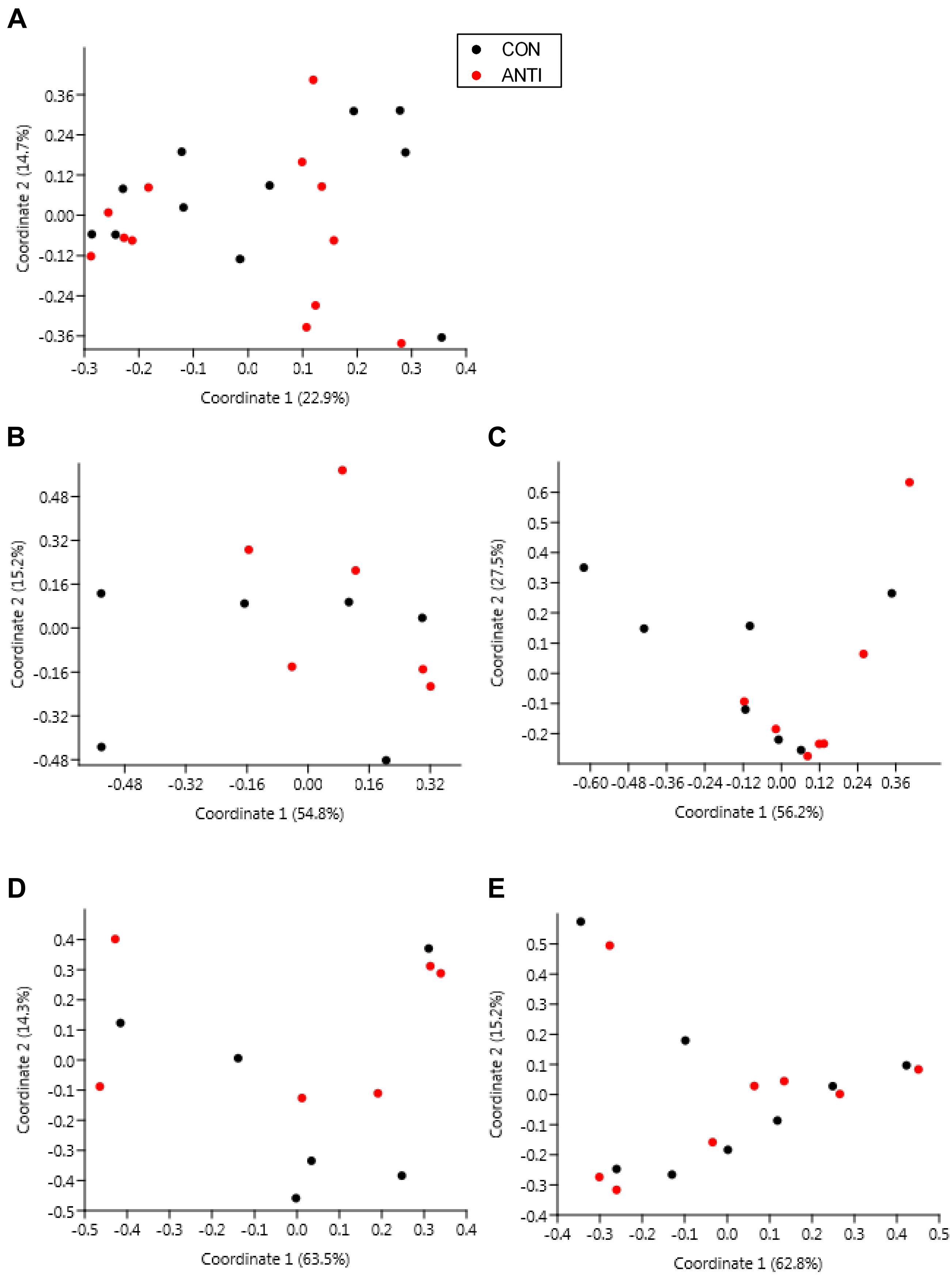
A
CON
ANTI
Coordinate 2 (14.7%)
0.36
0.24
0.12
0.00
-0.12
-0.24
-0.36
-0.3 -0.2 -0.1 0.0 0.1 0.2 0.3 0.4
Coordinate 1 (22.9%)
B
Coordinate 2 (15.2%)
0.48
0.32
0.16
0.00
-0.16
-0.32
-0.48
-0.48 -0.32 -0.16 0.00 0.16 0.32
Coordinate 1 (54.8%)
C
Coordinate 2 (27.5%)
0.6
0.5
0.4
0.3
0.2
0.1
0.0
-0.1
-0.2
-0.60 -0.48 -0.36 -0.24 -0.12 0.00 0.12 0.24 0.36
Coordinate 1 (56.2%)
D
Coordinate 2 (14.3%)
0.4
0.3
0.2
0.1
0.0
-0.1
-0.2
-0.3
-0.4
-0.5
-0.5 -0.4 -0.3 -0.2 -0.1 0.0 0.1 0.2 0.3 0.4
Coordinate 1 (63.5%)
E
Coordinate 2 (15.2%)
0.5
0.4
0.3
0.2
0.1
0.0
-0.1
-0.2
-0.3
-0.4
-0.4 -0.3 -0.2 -0.1 0.0 0.1 0.2 0.3 0.4 0.5
Coordinate 1 (62.8%)

**Supplemental Figure S2. PCoA plots of bacterial community composition.**

Bacterial community composition of (**A**) PND 7 rectal swabs, (**B**) PND 21 distal colon, (**C**) PND 21 ileum, (**D**) PND 49 distal colon, and (**E**) PND 49 ileum were shown in PCoA plots using Bray-Curtis dissimilarity metrics in amoxicillin treated (ANTI, red) and control (CON, black) pigs. All OTUs were retained in the analysis.

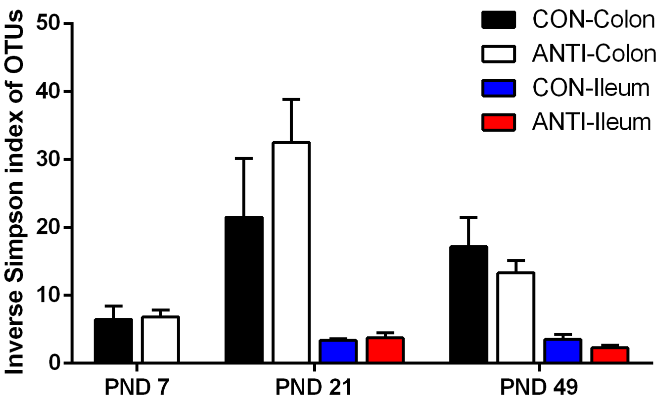


**Supplemental Figure S3. Effect of antibiotic treatment on alpha diversity of gut microbiota.**

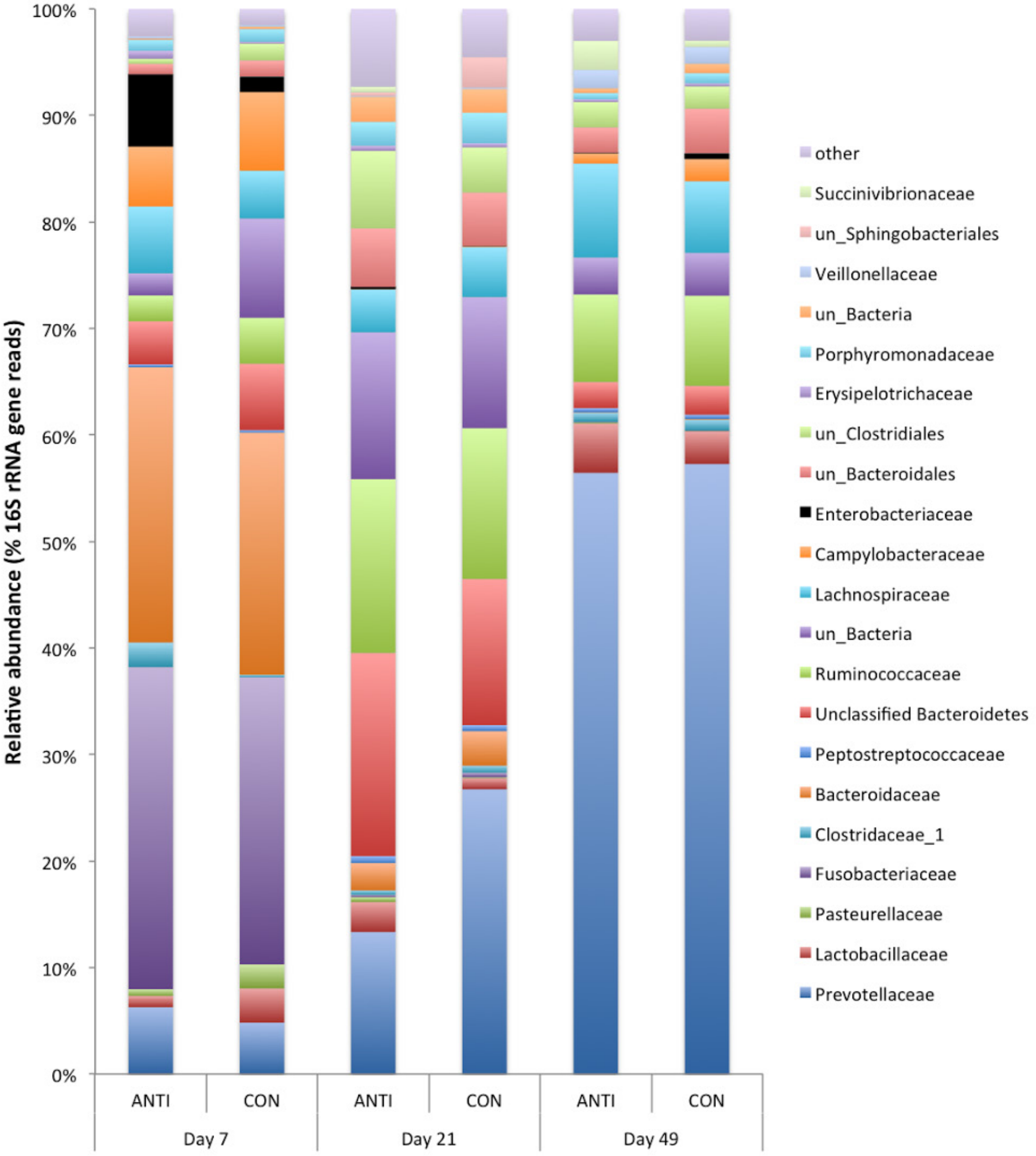


**Supplemental Figure S4 Proportion of bacterial families in distal colon samples**. Mean relative proportion of bacterial families in distal colon samples of piglets treated with amoxicillin (ANTI) or placebo (CON) for first two weeks of life at postnatal day 7, 21 and 49. Enrichment of Enterobacteriaceae in ANTI pigs is only observed during antibiotic treatment at day 7 ($P < 0.05$).

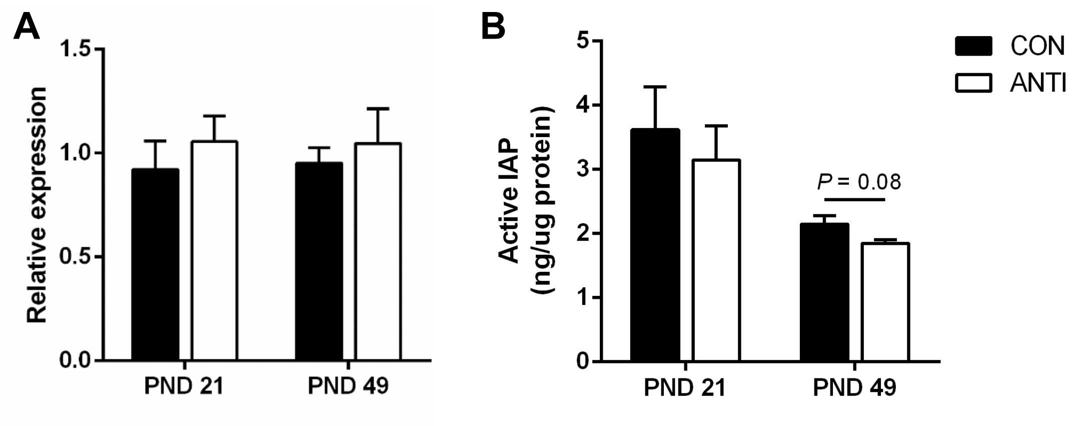


**Supplemental Figure S5. Intestinal alkaline phosphatase (IAP) gene expression levels and activity.**

The relative gene expression (**A**) and activity (**B**) of IAP at PND 21 and PND 49 were measured in ileum.

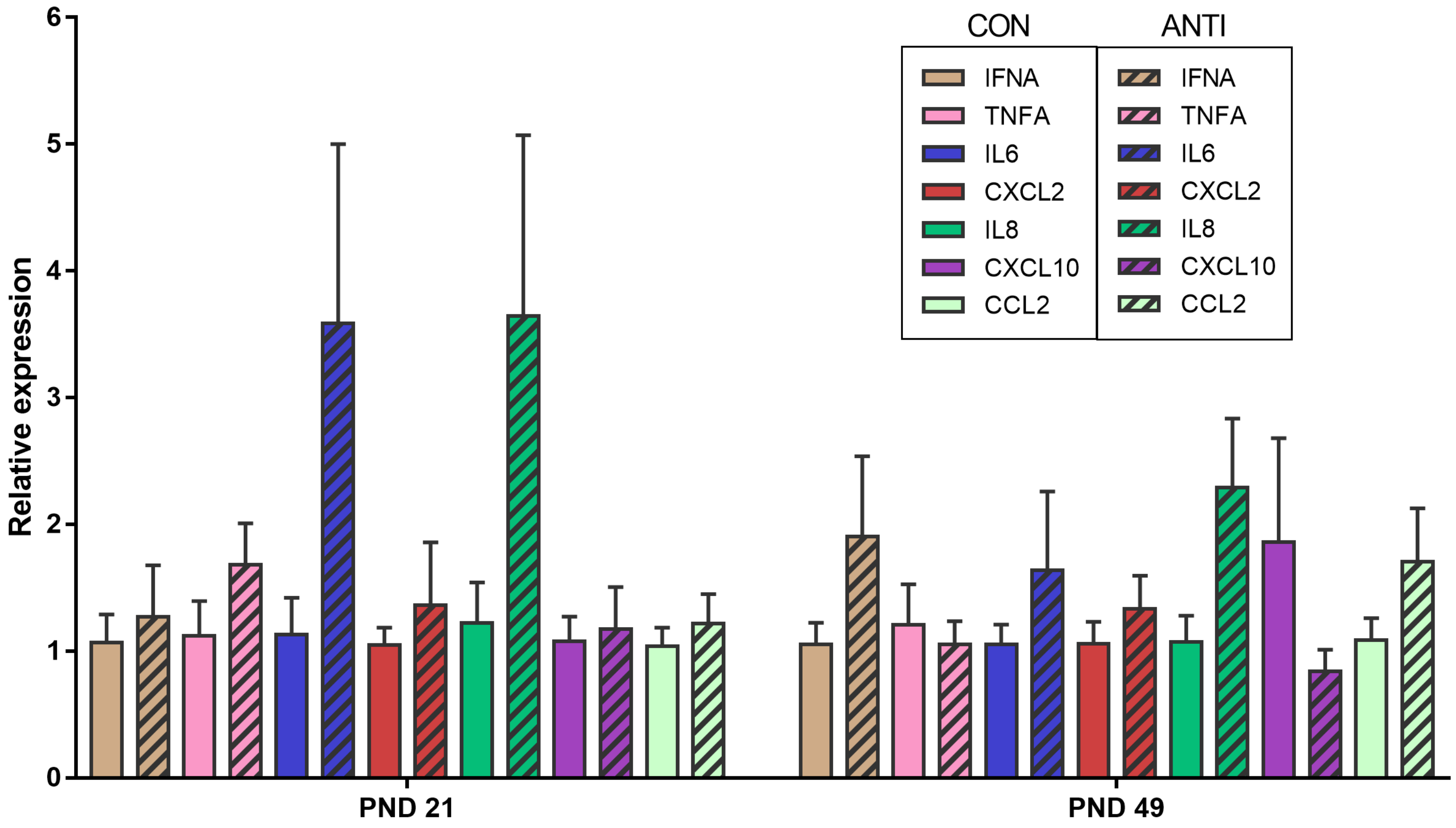


**Supplemental Figure S6. Pancreatic gene expression of cytokines and chemokines.**

The relative gene expression of IFNα, TNFα, IL6, CXCL2, IL8, CXCL10, and CCL2 at PND 21 and PND 49 were measured in whole pancreas.

## Supplemental Tables

### Supplemental Table S1. Ingredients and chemical composition of the piglet diets

| | Diet [a] (g/kg) | | | |
|---|---|---|---|---|
| Ingredients | Phase 1 | Phase 2 | Phase 3 | Pre-grower |
| Wheat, ground | 270.3 | 463.8 | 599.7 | 508.4 |
| Soybean meal, 460g CP/kg | 138.3 | 168 | 123 | 124 |
| Canola meal | 0 | 0 | 0 | 55 |
| Peas, ground | 0 | 0 | 0 | 75 |
| HI-Lactose whey | 171.2 | 125 | 0 | 0 |
| Distillers wheat husky | 0 | 30 | 50 | 0 |
| Corn | 0 | 0 | 94 | 150 |
| Oat | 220 | 100 | 59 | 0 |
| Fish meal, Menhaden 620g CP/kg | 60 | 37.5 | 0 | 0 |
| Fat | 15 | 40 | 25 | 18 |
| Canola oil | 10 | 0 | 0 | 0 |
| Limestone / glass rock | 8.5 | 15 | 15 | 19.2 |
| Mono/dicalcium phosphate | 0 | 0 | 4.3 | 0 |
| Salt | 0 | 0.5 | 4.7 | 3.9 |
| L-Lysine HCl 780g/kg | 4.3 | 5.2 | 8.3 | 4.4 |
| L-Threonine 990g/kg | 0.8 | 2.2 | 3.3 | 1.5 |
| DL-Methionine 990g/kg | 1.9 | 2 | 2.7 | 1.2 |
| L-Tryptophan 990g/kg | 0 | 0.1 | 0.4 | 0 |
| Animal plasma 920 | 35 | 0 | 0 | 0 |
| Water-glycol blend | 35 | 0 | 0 | 0 |
| Proform pig starter premix | 25 | 0 | 0 | 0 |
| extraPRO™ | 0 | 0 | 0 | 35 |
| Others | 4.7 | 10.7 | 10.6 | 4.4 |
| Dry matter (%) | 87.6 | 89.7 | 88.8 | 89.5 |
| Digestible energy (kcal/kg) | 3577 | 3627 | 3533 | 3483 |
| Metabolizable energy (kcal/kg) | 3385 | 3403 | 3337 | 3268 |
| Net energy (kcal/kg) | 2463 | 2622 | 2500 | 2349 |
| Crude protein (%) | 22.8 | 20.2 | 18.1 | 21.5 |
| Crude fat (%) | 5.0 | 5.8 | 4.3 | 5.4 |
| Lysine (%) | 1.64 | 1.36 | 1.30 | 1.30 |
| Threonine (%) | 1.00 | 0.91 | 0.87 | 0.92 |
| Methionine (%) | 0.54 | 0.49 | 0.48 | 0.43 |
| Tryptophan (%) | 0.30 | 0.24 | 0.24 | 0.25 |

**a** Phase 1, 2,3 and pre-grower diets are standard feed pallets produced by HI-PRO FEEDS, Sherwood Park, AB.

**Supplemental Table S2 Porcine-specific primer sequences used in quantitative RT-PCR analysis**

| Gene | Accession No. | Primer | Primer sequence (5'→ 3') | Ta (°C) | Amplicon length |
|---|---|---|---|---|---|
| ***IGF2*** | NM_213883.2 | Forward | AGGGCATCCAAACCACAAAC | 60 | 96 bp |
| | | Reverse | GGGTTCAATTTTTGGTATGTAACTTG | | |
| ***INS-2*** | NM_001109772.1 | Forward | CAGAAGCGTGGCATCGTG | 60 | 76 bp |
| | | Reverse | CCTAGTTGCAGTAGTTCTCCAG | | |
| ***PDX-1*** | NM_001141984.1 | Forward | AAGTCTACCAAGGCTCACGC | 60 | 159 bp |
| | | Reverse | GCGCGGCCTAGAGATGTATT | | |
| ***GPR41*** | XM_005664489.1 | Forward | TGGAGACCTTACGTGTTG | 57 | 75 bp |
| ***(FFAR3)*** | | Reverse | CGAGGATGAGAAGTAGTAGAT | | |
| ***GPR43*** | NM_001278758.1 | Forward | CGTGTTCATCGTTCAGTA | 57 | 76 bp |
| ***(FFAR2)*** | | Reverse | GAAGTTCTCATAGCAGGTA | | |
| ***IAP*** | XM_003133729.2 | Forward | CTAAAGGGGCAGATGAATGG | 60 | 105 bp |
| | | Reverse | CACCTGTCTGTCCACGTTGT | | |
| ***TNFa*** | X57321.1 | Forward | CACCACGCTCTTCTGCCTACTGC | 60 | 162 bp |
| | | Reverse | TCGGCTTTGACATTGGCTACAA | | |
| ***IFNa*** | M28623 | Forward | GGACCTGGAAGCCTGTGTCA | 60 | 326 bp |
| | | Reverse | CACGGGCAGGATGATACAGAAA | | |
| ***IL6*** | M86722.1 | Forward | GCCCACCAGGAACGAAAG A | 60 | 109 bp |
| | | Reverse | AAGCAGCCCCAGGGAGAA | | |
| ***IL8*** | NM_213867 | Forward | CCGTGTCAACATGACTTCCAA | 60 | 74 bp |
| | | Reverse | GCCTCACAGAGAGCTGCAGAA | | |
| ***CCL2*** | NM_214214 | Forward | GCGGCTGATGAGCTACAGAAG | 60 | 74 bp |
| | (USDA) | Reverse | CCGCGATGGTCTTGAAGATC | | |
| ***CXCL2*** | NM_001001861 | Forward | CCGTGCAAGGAATTCACCTC | 60 | 125 bp |
| | | Reverse | TGCGGGGTTGAGACAAACTT | | |
| ***CXCL10*** | NM_001008691 | Forward | CCCACATGTTGAGATCATTGC | 60 | 168 bp |
| | | Reverse | CATCCTTATCAGTAGTGCCG | | |
| ***GAPDH*** | NM_001206359.1 | Forward | CACCTGTCTGTCCACGTTGT | 60 | 147 bp |
| | | Reverse | ATGGACCGTGGTCATGAGT | | |

Ta: annealing temperature
